# Assessing impending hazards from summit eruptions: the new probabilistic map for lava flow inundation at Mt. Etna

**DOI:** 10.1038/s41598-023-46495-0

**Published:** 2023-11-09

**Authors:** Francesco Zuccarello, Giuseppe Bilotta, Gaetana Ganci, Cristina Proietti, Annalisa Cappello

**Affiliations:** Istituto Nazionale di Geofisica e Vulcanologia, Osservatorio Etneo, 95125 Catania, Italy

**Keywords:** Natural hazards, Volcanology

## Abstract

The development of probabilistic maps associated with lava flow inundation is essential to assess hazard in open vent volcanoes, especially those that have highly urbanized flanks. In this study we present the new lava flow hazard map linked to the summit eruptions of Mt. Etna, which has been developed using a probabilistic approach that integrates statistical analyses of the volcanological historical data with numerical simulations of lava flows. The statistical analysis of volcanological data (including vent location, duration and lava volumes) about all summit eruptions occurred since 1998 has allowed us both to estimate the spatiotemporal probability of future vent opening and to extract the effusion rate curves for lava flow modelling. Numerical simulations were run using the GPUFLOW model on a 2022 Digital Surface Model derived from optical satellite images. The probabilistic approach has been validated through a back-analysis by calculating the fit between the expected probabilities of inundation and the lava flows actually emplaced during the 2020-2022 period. The obtained map shows a very high probability of inundation of lava flows emitted at vents linked to the South East Crater, according to the observation of the eruptive dynamics in the last decades.

## Introduction

Open-conduit basaltic volcanoes are known to be a constant threat for the population living nearby them, since emission of volcanic products (e.g. lava flows, tephra fall-out, pyroclastic density currents) occur frequently due to their almost-persistent activity, which potentially may impact human infrastructures causing significant economic damages and, in some cases, even fatalities^1–5^. Assessment of the volcanic hazard linked to the various volcanic phenomena in open-conduit volcanoes is critical to reduce economic and human loss^6,7^. Even though highly energetic explosive events have been observed in these volcanoes^8–10^, lava flows represent the most common hazard for the human buildings, especially in those areas where the expansion of the urbanization led to an increase of their exposure. In this regard, long-term hazard linked to lava flows can be assessed through a qualitative analysis of historical eruptions^11,12^ or combining the volcanological record with deterministic and/or probabilistic computational models^13–15^. The latter can be used to develop probabilistic maps that outline the areas with the highest probabilities of inundation within a defined time span, representing a useful tool for land use planning and to support mitigation strategies during volcanic emergencies. The hazard maps can be obtained by integrating a robust approach to calculate the probabilities of occurrence of the expected events with numerical modeling that allows to simulate lava flows and to define their most likely paths^16,17^. Among the parameters required to run lava flow simulations, effusion rates strongly control the run-out of lava flows emplacements^18^, with important repercussions on the final computed probabilities. Indeed, different distributions of the effusion rates over time impact the final emplacement of lava flows^19^. Therefore, an accurate characterization of the effusion rate trends consistent with the observed time-averaged discharge rates (TADR) is fundamental to develop reliable hazard maps.

One of the most active open-conduit volcanoes in a very populated area is represented by Mt. Etna, which frequently emits lava flows from both the summit and from lateral fissures on the flank of the volcanic edifice^1^. Even though flank eruptions are more threatening than summit activity for the inhabited areas, summit eruptions are more frequent and may cause damages to the touristic facilities localized on the higher part of the volcano. Indeed, a general increase of the eruptive rate has been characterizing the current eruptive cycle at the main summit craters since the middle of the last century^20^. Several series of short-lasting paroxysmal eruptions, with high rates of lava emission^21–23^, have been alternated with long-lasting eruptions, with lower rates of lava emission. These long-lasting eruptions occur at the main craters or from vents located close to the summit that are fed directly by the central conduits, known as sub-terminal eruptions^24,25^.

**Figure 1:**
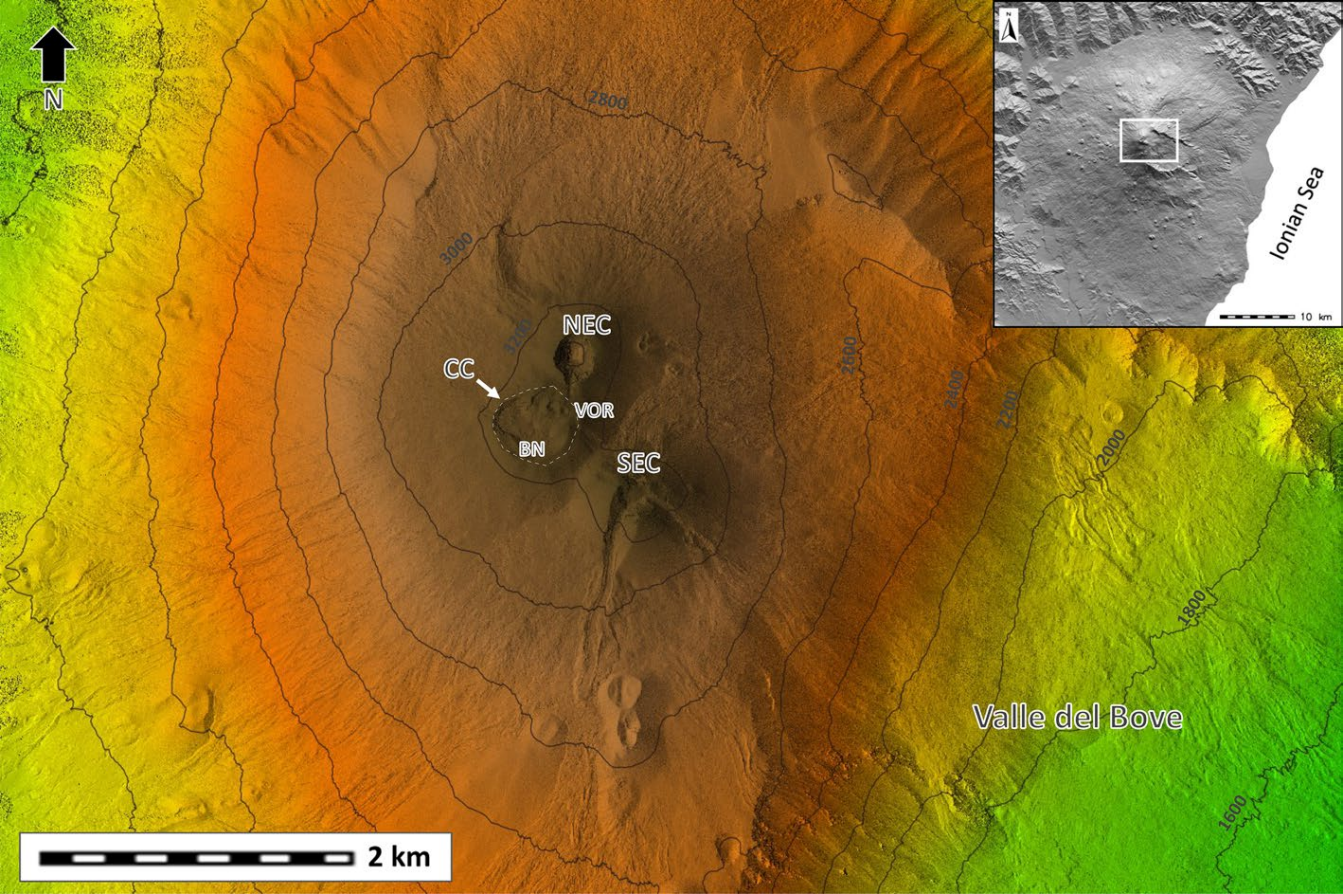
Map of the Mt. Etna summit (white rectangle in the inset), showing the location of the summit craters: North East Crater (NEC), South East Crater (SEC), Voragine (VOR) and Bocca Nuova (BN), which are part of the Central Crater (CC). Valle del Bove is a large horseshoe-shaped depression located eastward the summit craters. This figure was generated using the free and open source GRASS GIS software.

The recent 2020–2022 activity^26^, which has caused modifications of the topography due to the fast emplacements of volcanic products, has posed the need to to review and update the hazard assessment process linked to lava flows, considering shorter forecast periods and the change in the eruptive style of the volcano^15,17^. In this study we present a new hazard map associated to lava flows inundation at the summit of Mt. Etna volcano by using a probabilistic approach that considers both the long-lasting sub-terminal eruptions, and the short-lasting paroxysmal events from the summit craters: North East Crater (NEC), South East Crater (SEC), Voragine (VOR) and Bocca Nuova (BN), the latter considered as part of the complex representing the old Central Crater (CC) (Fig. 1).

The study also emphasizes the importance of model validation through a back-analysis, which ensures the reliability of hazard maps. To this end, we compare the probability of inundation computed for the period 2020–2022 with the observed paths taken by lava flows in the same period. Two methods have been adopted to calculate the probabilities of occurrence of expected events, by assuming different probability distributions (i.e. Poissonian vs. Non-Poissonian), and the more suitable approach has been used to develop the new hazard map including the new events actually occurred until mid 2022, covering the next three years starting from 2023. The probabilistic approach, as well as the validation methodology, contribute to the novelty of the study, being designed to be applied also to other active volcanic areas, for which detailed volcanological historical data are available.

## Results

The methodology adopted in this study to build the hazard maps for both the back-analysis and post-2023 period combines the historical record of the volcanic activity with computational numerical models to simulate lava flows emplacement on a 5m-resolution Digital Surface Model (DSM), obtained from Pléiades imagery^26^. The methodology includes different stages: (i) the definition of the classes of the expected eruptions, effusion rate curves and their occurrence probability; (ii) assessment of the spatiotemporal probability of future vent opening, by assuming two different probability distributions; (iii) running of lava flows emplacements considering a large number of eruptive scenarios with the GPUFLOW model^27^; (iv) computation of the long-term probability for each point of the area interested by simulated lava flows; (v) validation of the best approach.

### Eruptive classes and effusion rate curves

In the last decades, the main site of both explosive and effusive activity at Etna’s summit is the area around the SEC^20^. This crater has produced more than 200 episodes of paroxysmal eruptions since 1998, where the last 62 episodes occurred during the recent paroxysmal 2020–2022 series^22^. These more recent events, for which we collected the main quantitative volcanological data (e.g. start date, duration of lava effusion and lava fountaining, lava volume, and cumulative volume), were added to the catalogue used for the previous summit hazard map^20^.

**Figure 2:**
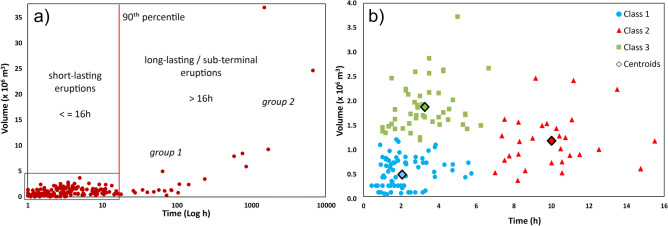
(**a**) Diagram showing the distribution of durations (in hours) and volumes (in million cubic meters) for all eruptions considered in our analysis, covering the 1998–2023 period. (**b**) Zoom-in of the previous diagram on the short-lasting eruptions and their classification.

**Figure 3:**
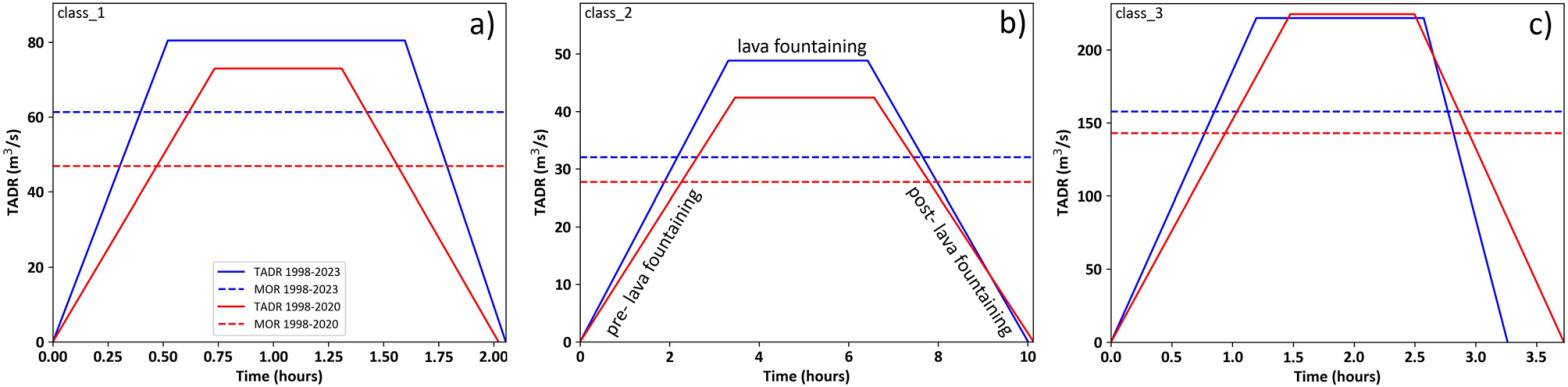
Time-averaged discharge rate (TADR) curves for the short-lasting eruptive classes extracted from the statistical analysis of past events considering the 1998-2020 and 1998-2023 periods ((**a**) class 1, (**b**) class 2, (**c**) class 3).

The statistical analysis provided that the 90th percentile of short-lasting eruptions show durations lower than 16h, representing paroxysmal eruptions characterized by high energy and output rates (Fig. 2a). Considering the dataset up to 2020, three eruptive classes linked to the short-lasting events were recognized and grouped (Fig. 2b): (i) *class 1*, represented by a duration of 2.02h and volume of lava erupted of $0.34 \times 10^6\,\text {m}^3$; (ii) *class 2*, represented by a duration of 10.1h and volume of lava erupted of $1.01 \times 10^6\,\text {m}^3$; (iii) *class 3*, represented by a duration of 3.73h and volume of lava erupted of $1.92 \times 10^6\,\text {m}^3$. Average MORs (mean output rates) were obtained by dividing the volumes of lava erupted per the average durations of eruptions, where values of ${\sim }47$, ${\sim }28$ and ${\sim }143\,\text {m}^3/\text {s}$ were obtained respectively for the three classes. The same calculation performed including the 2020–2022 period provided similar durations but slightly higher volumes, obtaining MOR values of ${\sim }61.$, ${\sim }32$, and ${\sim }158\,\text {m}^3/\text {s}$, respectively. Time-averaged discharge rate curves were built in order to be consistent with the observed eruptive dynamic to be used for the lava flow modeling. They show a trapezoidal shape characterized by a linear increase of the effusion rate during the pre-lava fountaining phase, followed by a constant value of effusion rate for the duration of the lava fountaining and then decreasing linearly until the end of the eruption (Fig. 3).

Long-lasting eruptions, characterized by mild Strombolian activity and low effusion rates of lava emitted from the main craters or from sub-terminal vents, and with duration greater than 16h, constitute separated classes. The distribution of durations and volumes of lava erupted linked to the these eruptions highlights at least two groups (Fig. 2a): (i) *group 1* shows a duration in the order of days to weeks and volume of lava erupted from 0.3 to $3.5 \times 10^6\,\text {m}^3$; (ii) *group 2* has a duration longer than a month and a volume from 5.9 to $9.3 \times 10^6\,\text {m}^3$. However, the normalization of the effusion rate curves highlighted two distinct trends for *group 1*. *Trend 1* shows a peak in effusion rate between 7 and 31% of the total duration (50th percentile of the peak at 21%), followed by an exponential decrease with the inflection points localized at  48% of the total duration and with a flux value of 23% of the peak. The effusion rate peaks in *trend 2* are localized between 30% and 70% of the total duration (50th percentile at 50%), with bell-shape curves or with a plateau at nearly constant effusion rate. Conversely, *group 2* is characterized by a progressive increase in effusion rate and reaching the peak between 66 and 81% (50th percentile at 79%). Therefore, the classes recognized for the long-lasting/sub-terminal eruptions are three: *class 4* and *class 5*, which are both represented by a duration of 4 days and volume of $1.5 \times 10^6\,\text {m}^3$, but with different trends of the effusion rate curves, and *class 6*, which is represented by a longer duration of 46 days and a greater volume of $7.9 \times 10^6\,\text {m}^3$.

**Figure 4:**
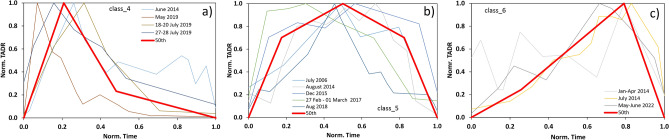
TADR curves for the long-lasting eruptive classes extracted from the statistical analysis of past events ((**a**) class 4, (**b**) class 5, (**c**) class 6). Durations and rates are normalized to the total duration and peak rates of the analyzed events.

The effusion rate curves for *class 4* and *class 6* were built by calculating the 50th percentiles of the peaks and inflection points occurrence. For *class 5*, we used an averaged value of the 50th percentiles linked to the inflection points pre- and post-peak to build a symmetrical bell-shaped curve (Fig. 4).

The frequency of events from 1998 until 2020 suggests that the eruptive *class 1* is the most frequent (50%), followed by *class 2* (21%) and *class 3* (14%), with the remaining 15% distributed approximately equally among the eruptive *classes 4, 5* and *6* associated to the long-lasting eruptions (5% each). Minor differences have been observed if we include the data of the 2020–2022 activity: *class 1*: 49%, *class 2*: 19% *class 3*: 21%, *class 4, 5, 6*: slightly less than 4% each. Since only the May-June 2022 eruption has been considered in the analysis for the post-2020 period, we used the same effusion rate curves to simulate lava flows emplacements both for the back-analysis on the pre-2020 topography and for the new map on the 2022 topography^26^.

### Spatiotemporal probabilities of future vent opening

For the estimation of the spatiotemporal probability of future vent opening, we again separately considered the short- and long-lasting eruptions characterising the summit area. Indeed, during paroxysmal activity, lava overflows occur chiefly from the main vents or from minor vents on the upper flanks of the summit craters, whereas long-lasting eruptions may occur also on sub-terminal fractures at hundreds of meters from the main vents.

**Figure 5:**
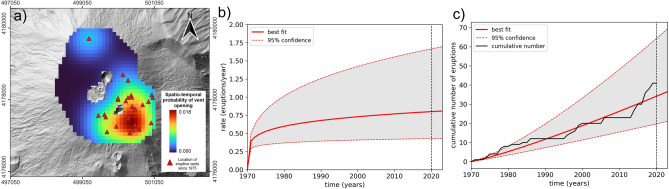
(**a**) Spatiotemporal probability of future vent opening for the period 2020–2022 for long-lasting sub-terminal eruptions (red triangles: locations of known vents associated to past events since 1970; this figure was generated using the free and open source QGIS software); (**b**) diagram showing the variation in the time of the annual eruptive rate, calculated by fitting the observed cumulative number of the long-lasting eruptions since 1970 (black line in panel (**c**)).

Despite a good documentation of the historical records of the past century linked to the whole summit activity (Ref.^28^ and references therein), accurate information on the vents location are not available before 1970. Therefore, we calculated the spatial distribution of opening of future sub-terminal vents through the probabilistic approach (see "Methods" section) considering only the last five decades, where most of the vents are located close to the SEC area (Fig. 5a). The eruptive rate has been calculated by considering a Poissonian distribution of the long-lasting eruptions occurred since 1970, and extending the expected rate to the next 3 years (Fig. 5b,c). The calculation was performed for both 1998–2020 and the whole period including the May-June 2022 eruption, to obtain respectively a map covering the 2020–2022 period for back-analysis, and a post-2023 map as the final product. The spatial and temporal data are combined in order to generate the spatiotemporal probability map of new vent opening for the chosen time interval. The obtained maps do not show substantial differences and the southeast area of SEC is characterized by the highest probability of future vent opening linked to the long-lasting eruptions.

**Figure 6:**
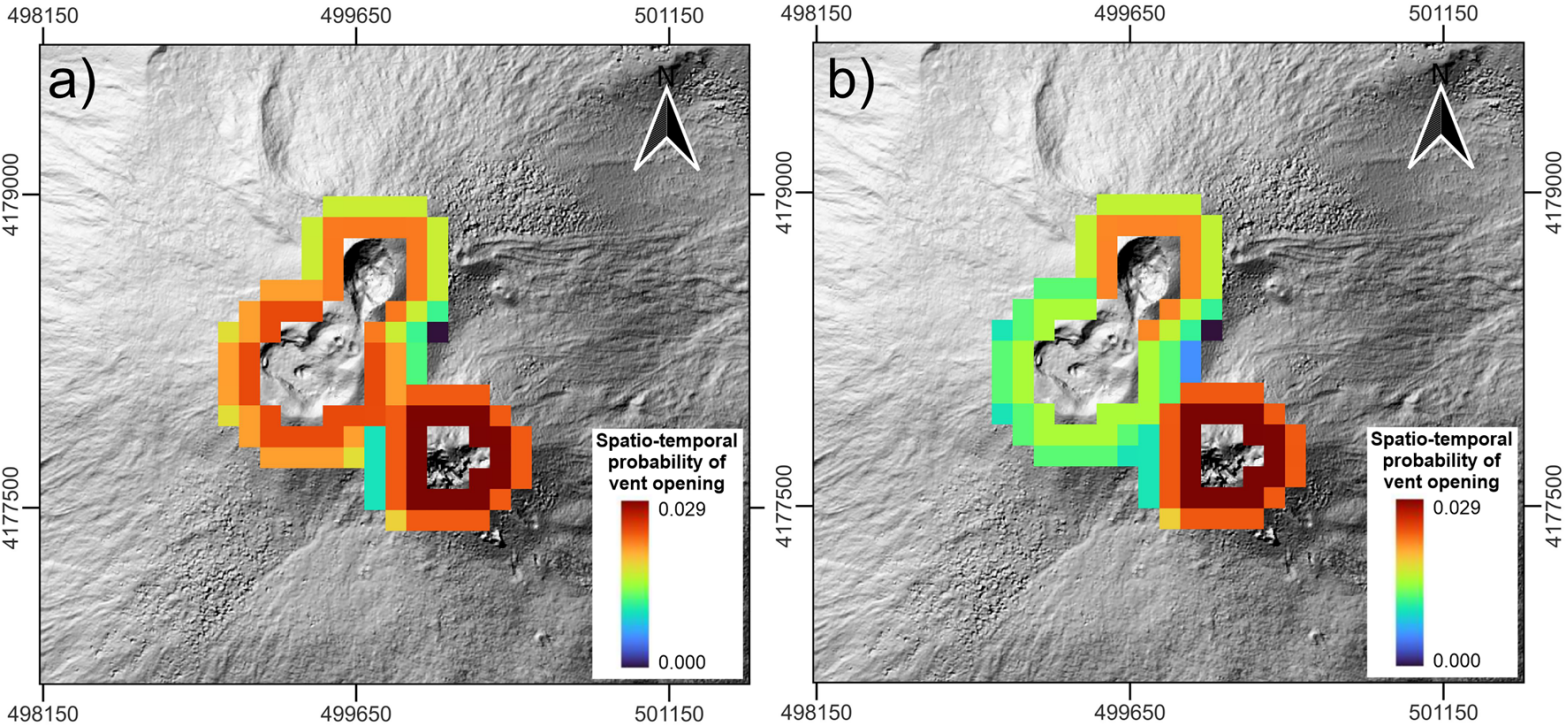
Spatiotemporal probability of future vent opening for the period 2020–2022 for short-lasting eruptions: (**a**) assuming a Poissonian distribution of the eruptive events; (**b**) through DTMC. This figure was generated using the free and open source QGIS software.

For short-lasting events represented by the paroxysmal eruptions, we first calculated the temporal probabilities of occurrence of events for each crater, since this type of eruptions affect the same craters several times during their history. For the back-analysis, we tested two different approaches to calculate the temporal probability. The first attempt asssumes a Poissonian distribution of the paroxymal episodes, calculating the probability as a function of the annual eruptive rate for each crater. However, in the last 40 years, paroxysmal eruptions occurred mostly in series of several episodes over a time span from weeks to months^29^. This reflects that the probability of occurrence of a single episode is greater within a series than in the time intervals between two series, suggesting that the lava fountaining episodes do not occur following a Poissonian distribution. So, in the second approach we used a Discrete-Time Markov Chain (DTMC) to estimate the temporal probability of moving to a lava fountaining state from any individual state (i.e. quiescence, persistent Strombolian activity, effusive activity from main vents or from sub-terminal vents). The calculation has been performed for each summit crater considering the eruptive record since the end of the 1991–1993 eruption, for which a better knowledge of the daily status for each crater is available from literature^28^ and the periodic bullettins emitted by INGV. Then, we have distributed the total probability uniformly on the area around each interested crater, considering a distance from the crater rims of 150–300m, where the highest values are localized around the SEC (Fig. 6a,b).

### Lava flow simulations and development of the hazard maps

A regular grid with 100m spacing in both directions has been overlaid on the summit area, with each intersection counting as a potential vent. For the areas closer to the summit, simulations for each of the 6 eruptive classes have been run, whereas only the three classes linked to the long-lasting eruptions were considered at the potential vents external to the summit craters. The lava flow simulations were modeled through GPUFLOW^27^ using as input the effusion rate curves developed for each eruptive class. Two sets of simulations were performed using two different DSMs derived from Pléiades imagery with a resolution of 5m^26^: in the first, the DSM represents the topography on August 2020 and was used to build the hazard map for the subsequent three years (2020–2022), for back-analysis purposes; in the second set of simulations, the DSM representative of the topography on July 2022 was used to build the post-2023 hazard map. The probability of invasion at each point was computed by weighting each simulation covering that point by the spatiotemporal probability of the corresponding class and vent.

**Figure 7:**
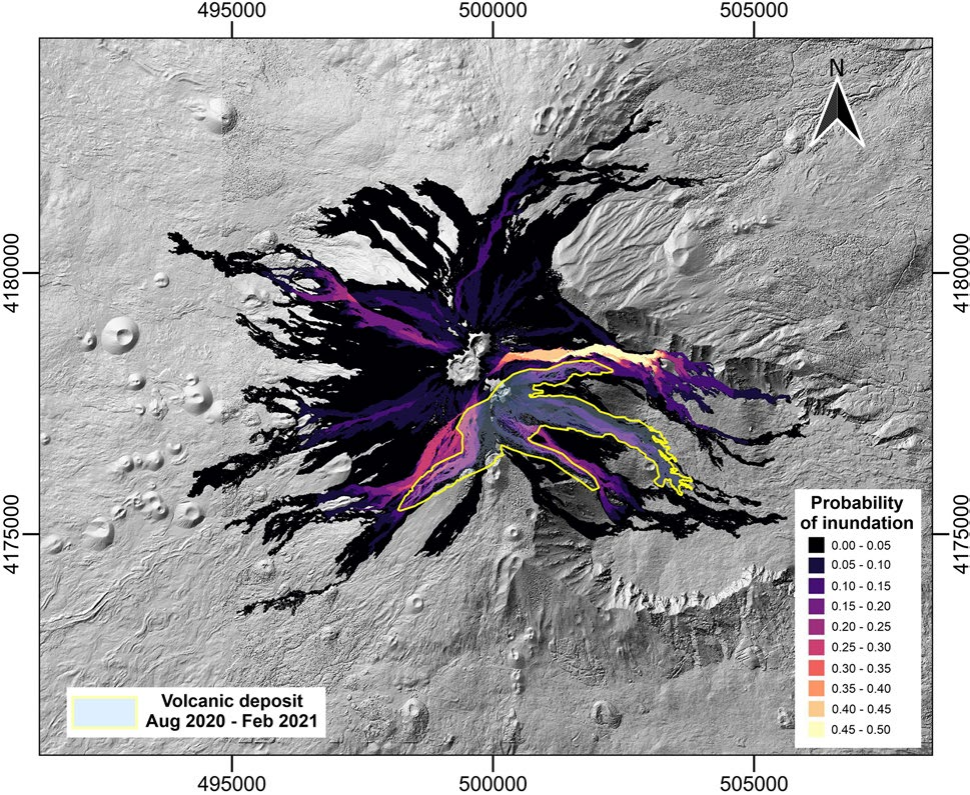
Summit hazard map for the 2020–2022 period, used for back-analysis, showing the outlines of the observed emplacements of volcanic products of the actual events that occurred in this period^26^. This figure was generated using the free and open source QGIS software.

**Figure 8:**
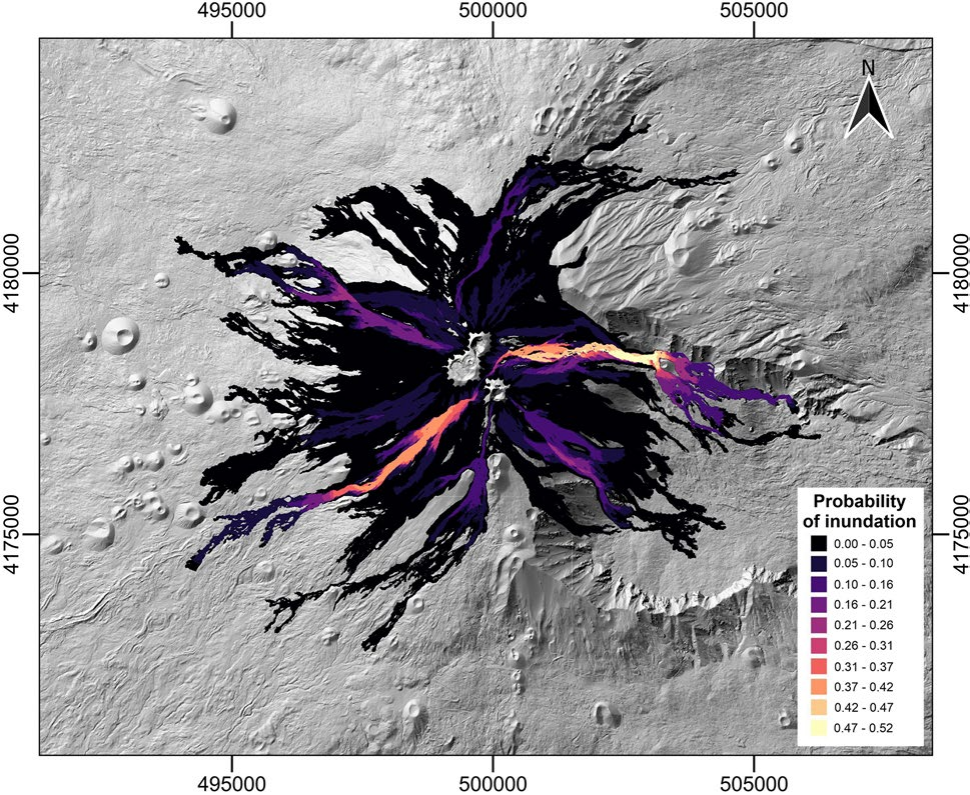
Post-2023 summit hazard map at Mt. Etna. This figure was generated using the free and open source QGIS software.

To determine the best choice of spatiotemporal probability for the short-lasting events, the back-analysis was conducted using the 2020–2022 hazard map computed both with the Poisson and DTMC methods. For the validation, we quantified the fit between the obtained probabilities and the volcanic deposit emplaced during the first eruptive period (August 2020–February 2021), that localized at east-north to south-west area of the SEC. Despite the results being very similar, the one obtained using the DTMC approach provided a slightly better fit (Fig. 7). The DTMC method was therefore used to calculate the temporal probability and to build the new hazard map for the next three years starting from the 2023 (Fig. 8). The obtained map shows a similar distribution of the probabilities as observed in the 2020–2022 map, where the highest probabilities are localized in direction of the Valle del Bove, a huge depression on the east flank of the volcano, and from south-east to south west sectors from the SEC. A relative high probability is also observed on the north-west flank, consistent with the lava flow paths observed during the May 2016 eruption at CC^30^.

## Discussion

The new lava flows hazard maps for the summit of Mt. Etna have been obtained using a different approach compared to the one used for the previous map^15^, particularly in the classification of events and the calculation of their probability. Additionally, the previous map was obtained considering a forecast period of 10 years, which is too long for the summit of Mt. Etna, being characterized by almost persistent activity. In fact, this behaviour results in substantial changes in the morphology of the summit area, implying that the DSMs used for simulations do not adequately represent the topography for too long time intervals. A shorter forecast period allowed us to calculate more accurate probabilities of occurrence of eruptive episodes linked to each recognized eruptive class, resulting in more reliable lava inundation probabilities of the interested areas. Moreover, the more refined characterization of the eruptive classes and effusion rate curves led to an improved distributions of the most likely paths taken by simulated lava flows, consistent with the observed paths in the last 10 years, where the maximum lengths are on the range of 4–7 km from the main vents^31^.

The two hazard maps associated to the 2020–2022 (Fig. 7) and post-2023 (Fig. 8) periods show that the simulated lava flow paths strongly control the distribution of the probabilities, which is maximal in those areas affected by a larger number of simulated lava flows. On the other hand, the higher temporal probabilities of occurrence for both short- and long-lasting eruptions at SEC compared to CC and NEC contribute to a slightly higher probabilities of inundation in a wider area in the east, southeast and southwest sectors of the volcano. This is consistent with the observed emplacement of volcanic products between August 2020 and February 2021, that shows a good fit with the hazard map associated to the 2020–2022 period (Fig. 7).

It is worth to note that the computed temporal probabilities for short-lasting eruptions have been distributed uniformly around each crater without considering any factor which may affect the distributions of the vent opening or lava overflow probabilities, such as the morphology of the crater or structural systems. Furthermore, partial collapses of the crater induced by the accumulation of pyroclastic deposit may occur during an ongoing eruption^32^, leading to a drastic change of the morphology of the crater and affecting the lava overflow dynamics. Even though we observed frequently a shift of the active structures, especially at the SEC area^33^, we don’t have any clues to predict morphological changes of the crater induced by the potential collapse, so we preferred not to take these factors into account in the distribution of the spatiotemporal probability of occurrence of eruptions. Like the previous summit and flank maps^15^, the computed probabilities of inundation depend on the morphological data known at the time of production of the map. Multiple events occurring in the same areas will lower the accuracy of the computed probabilities in the affected areas for subsequent lava flows.

Similarly, the post-2023 map differs from the 2020–2022 map on the SEC area because of the morphological changes caused by the emplacements of the volcanic products in the last three years, affecting the paths of simulated lava flows, as well as because of the minor differences in the frequencies of the eruptive classes for the short-lasting eruptions and in the associated effusion rate values. Indeed, the higher effusion rate values obtained for the short-lasting eruptive classes including the 2020–2022 records contribute to slightly increase the run-out distances of the simulated lava flows. Finally, the increase of the frequency of events linked to the eruptive *class 3* highlights an intensification of the magnitude of the paroxysmal events in the last decades in terms of volume of lava erupted and MOR, contributing to raise the hazard linked to the paroxysmal events.

This study showed how the probabilistic approach can be validated to estimate accurate probabilities of lava inundation in a very active volcano through a back-analysis, combining numerical modeling and statistical analysis of the volcanological data. The more refined approach showed here can be also used in very active volcanoes characterized by a high-frequency of eruptive events in the time.

## Methods

### Definition of eruptive classes and effusion rate curves

Calculation of percentiles associated to the eruption durations provided that short-lasting events with duration lower than 16h represent the 90th percentile of the total events, with the 1998–2014 data from Refs.^28,31,34,35^, and the data for the 2020–2022 paroxysmal activity from Ref.^26^ (see the Supplementary Information). A detailed classification based on erupted volumes and durations of these short-lasting eruptions has been performed using the *k*-means function in MATLAB®. Average MORs were derived for the short-lasting classes using the average volume and duration obtained from the clustering. In order to be consistent with the observed eruptive dynamics, the effusion rate curves associated to the short-lasting eruptions were built by fixing three time intervals: (i) the elapsed time between the beginning of lava effusion and the beginning of lava fountaining; (ii) the total duration of the lava fountaining phase; (iii) the elapsed time between the ending of the lava fountaining phase and the ending of the eruptive episode, assuming that the lava effusion stop simultaneously with the explosive activity. The maximum effusion rate at the lava fountaining phase for each class is fixed so that the integral volume over time matches the average value obtained from clustering (normalization). The time intervals were defined by the observation of the eruptive activity through thermal cameras for the 2011–2013 and 2020–2022 paroxysmal series, normalizing the values with respect to the total duration of the eruptive episodes and calculating the averaged values for each time interval. Then, the normalized values have been converted in theoretical duration and effusion rate values by using the average quantities obtained from the clustering.

For the long-lasting/sub-terminal eruptions, average values of erupted volumes and durations were calculated considering the post-2006 eruptive events, for which time-averaged discharge rates (TADRs) are available, following the method described in Ref.^19^. All TADR series were obtained from the conversion of infrared radiation collected by satellite measurements, using SEVIRI (January–April 2014, June 2014, July–August 2014, December 2015 eruptions data from Ref.^31^; 2017 eruption data from Ref.^36^; August 2018, May 2019, July 2019 and May–June 2022 eruptions data this study; see the Supplementary Information), and MODIS^16^ (July 2006 eruption). The curves were firstly filtered to remove the high-frequency noise and convoluted by selecting local positive peaks. Then, duration and flux values were normalized by dividing each sampled point by the respective maximum values to obtain homogeneous curves^19^. Analysis on the distribution of effusion rate peaks and inflection points allowed us to the define the three classes presented earlier and to build characteristic curves consistent with the observed estimated from satellite for each recognized eruptive class.

The obtained effusion rate curves have been used to simulate lava flows using GPUFLOW^27^, a 2D cellular automaton that improves over the MAGFLOW model^37^ used to build the previous hazard maps^15,17^. In addition to the effusion rate curves, the physical properties of the fluid (e.g. melt compositions, water content, rheological law, thermal properties) and the topography of the terrain, given as DSM, constitute input parameters required by the model. The lava flows modeled to develop the new hazard map at Mt. Etna were simulated by using average values of the physical properties of lava flows (i.e. density: $2600\,\text {kg/m}^3$, water content: 0.05 wt.%; initial temperature: 1360 K, solidus temperature: 1147 K; Ref.^17^), on the most recent DSM, representing the topography of Mt. Etna in July 2022 for the final product, and the 2020 topography for the back-analysis.

### Probabilistic model for the assessment of lava flow hazard

To develop the hazard map at Mt. Etna summit, we used a probabilistic model. For the short-lasting eruptions, a 100m space grid of potential vents $v_i(x,y)$ around the summit craters has been defined, covering a distance of 150–300m from the rims. The grid of potential vents linked to the long-lasting/sub-terminal eruptions was extended to the whole area corresponding to the Ellittico caldera, formed after a series of strong explosive events during the final phase of the Ellittico volcano 15ky ago^38^.

In the case of long-lasting/sub-terminal eruptions, a probability density function (PDF) was calculated starting from the eruptive fissures from previous events, with the influence on the potential vents computed using a 2D Gaussian kernel:

1 $$\begin{aligned} W_i(v_i, h_i) = \sum _{j=1}^{N} \left( \frac{1}{2\pi h^2} \exp {\left( -\frac{d_{ij}}{2h^2}\right) }\times \exp {(kt_j)} \right) \end{aligned}$$

where *N* is the number of the eruptive events since 1971 (when SEC was born), $d_{ij}$ is the distance between *i*-th potential vent and the *j*-th real vent, *h* is the smoothing factor, *k* is a constant that assigns larger weights to more recent events with respect to older ones, and $t_i$ is the time elapsed time since the eruption of the considered fissure. The smoothing factors *h* has been constrained using the Least Square Cross-Validation (LSCV), which minimize the integrated square error between the true and the estimated distribution of the vents^20^, providing a best estimate of 324m. The best fit for the constant *k* has been obtained by calculating the Brier Score (BS)^39^, providing a value of 0.035 (smaller values close to 0 indicating better forecasts). The probability of at least a new vent opening in the unit area $\Delta {x}\Delta {y}$ ($100\,\text {m}\times 100\,\text {m}$) within time $\Delta {t}$ is calculated with the nonhomogenous Poisson process (NHPP) model as follows:

2 $$\begin{aligned} p_a(x,y,\Delta {t}) = 1 - \exp {(\lambda {_{xyt}} \Delta {x} \Delta {y} \Delta {t}}) \end{aligned}$$

where $\lambda _{xyt}$ represents $W_i$ rescaled for the expected annual rate of eruptive events in the $\Delta t$ time span (3 years in our case). The expected time annual rate of eruptive events has been derived using the power intensity function (Ref.^40^ and references therein):

3 $$\begin{aligned} \lambda (t) = \frac{\delta }{\theta } \left( \frac{t-T_1}{\theta }\right) ^{\delta - 1} \end{aligned}$$

where $T_1$ is the initial year of the considered period (1970), *t* is the year of interest and assumes values ${} \ge T_1$, $\delta$ and $\theta$ are respectively the power coefficient and the scale parameter, which are unknown. The $\delta$ parameter determines the shape of the function: if $\delta = 1$, the rate is constant, otherwise the rate increase or decrease when $\delta > 1$ or $\delta < 1$. The best estimates of $\delta$ and $\theta$ are found through the least square minimization between the expected amount and the actual cumulated number of eruptions over time. The expected annual eruption rates for the considered forecast periods are between 1.86 and 2.00 eruption per year.

For the short-lasting events represented by paroxysmal eruptions, the summit area was divided by the three summit craters. In the first approach we calculated the temporal probabilities using the NHPP model as a function of the annual eruptive rate of occurrence of lava fountaining for each crater. In the second approach, the temporal probability of lava fountaining has been defined for each summit crater through the Discrete-Time Markov Chain (DTMC), by defining the transition matrix *M*:

**Table:** 

	$s_0$	$s_1$	$s_2$	$s_3$
$s_0$	$p_{00}$	$p_{01}$	$p_{02}$	$p_{03}$
$s_1$	$p_{10}$	$p_{11}$	$p_{12}$	$p_{13}$
$s_2$	$p_{20}$	$p_{21}$	$p_{22}$	$p_{23}$
$s_3$	$p_{30}$	$p_{31}$	$p_{32}$	$p_{33}$

which contains the daily probability $p_{ij}$, with $i,j = 0, 1, 2, 3$, of moving from the state $s_i$ to state $s_j$ ($s_0$: quiescence, $s_1$: persistent Strombolian activity, $s_2$: effusive activity from main vents or from sub-terminal vents, $s_3$: lava fountaining activity), considering the eruptive record from 1993 to 2020 (back-analysis) and then to 2023 (final map). Since we are interested in calculating the probability that at least one lava fountaining occurs at each summit crater, we compute a new transition matrix $M_s$ obtained from *M* by replacing the bottom row with [0, 0, 0, 1] to turn lava fountaining into an absorbing state. Then, we computed $M_s^n$ with $n=1096$ days (3 years), where the last column contains the probability to reach the lava fountaining state from any individual state. Finally, the total probability has been obtained by calculating the dot product between the vector with the initial probabilities $\pi$, fixing the lava fountaining state $s_3$ at 0, and the last column of $M_s^n$. Each obtained temporal probability has been distributed uniformly within the area up to 150–300m from the rims of the interested summit crater using the 2D Gaussian kernel, fixing the smoothing factor *h* as half of the radius of the crater.

The spatiotemporal probability of occurrence of the eruptive class *c* at each potential vent $v_i$ is then obtained by weighting $p_a$ for the $p_c$ probability of the eruptive class *c*, fixed as the relative frequency of occurrence of *c* respectively for both 1998–2020 and 1998–2023 periods:

4 $$\begin{aligned} p_{ac}(v_i,\Delta {t}) = p_a(v_i,\Delta {t}) \times p_c(v_i) \end{aligned}$$

Finally, the probability of lava inundation for each point (*x*, *y*) of the area covered by simulated lava flows is obtained by combining of the $p_{ac}$ spatiotemporal probability of vent opening for each eruptive class *c* with the overlapping lava flows generated by numerical simulations. We consider all the eruptive events independent and not mutually exclusive since the occurrence of an event does not influence the probability of occurrence of another event^15^. The total accumulation probability in the considered $\Delta t$ interval for each point (*x*, *y*) of the summit can be expressed as:

5 $$\begin{aligned} p(x,y,\Delta {t}) = 1 - \prod _{i=1}^{S_c(i)}(1 - p_{ac}(v_i,\Delta {t})) \end{aligned}$$

where $S_c(i)$ is the number of GPUFLOW simulations of the eruptive class *c* localized at vent $v_i$ inundating the point (*x*, *y*). The $p_{ac}$ linked to the short-lasting eruptions for each vent $v_i$ localized outside the summit area has been fixed at zero. In order to validate the model, we calculated the goodness of fit between the computed probabilities for the 2020–2022 and the observed volcanic deposit emplaced during the eruptive events between August 2020 and February 2021, comparing the BS linked to the two hazard maps obtained by using NHPP and DTMC for the short-lasting eruptions. A slightly lower value has been calculated for the map in which DTMC approach has been used, then validating this model to develop the new hazard map for the next three years starting from the 2023.

### Supplementary Information

Supplementary Information.

## Data Availability

All data generated or analysed during this study are included in this published article [and its supplementary information files].
